# Supplementary material for: Developmental Cascades Link Maternal–Newborn Skin-to-Skin Contact with Young Adults’ Psychological Symptoms, Oxytocin, and Immunity; Charting Mechanisms of Developmental Continuity from Birth to Adulthood
Source: Biology (Basel). 2023 Jun 13;12(6):847. doi: 10.3390/biology12060847 (PMC10295697; doi:10.3390/biology12060847)
Supplement: Supplementary file 1 [file biology-12-00847-s001.zip › biology-2391831-supplementary.pdf]

## Supplementary materials

Developmental Cascades Link Maternal-Newborn Skin-to-Skin Contact with Young Adults' Psychological Symptoms, Oxytocin, and Immunity; Charting Mechanisms of Developmental Continuity from Birth to Adulthood

Supplementary Table S1. Multiple regression predicting child's mental state

| | **Estimate** | **Std.error** | **t value** | **P** |
|---|---|---|---|---|
| **Group** | 0.06 | 0.19 | 0.33 | 0.74 |
| **birthweight** | -0.0001 | 0.0003 | -0.47 | 0.64 |
| **Mother age** | 0.04 | 0.02 | 1.84 | 0.072 |
| **Mother education (above average)** | 0.07 | 0.10 | 0.745 | 0.46 |
| **Household income** | -0.3 | 0.22 | -1.6 | 0.12 |
| **Father Education** | 0.17 | 0.10 | -1.76 | 0.084 |
| **Child's age** | -0.17 | 0.11 | -1.5 | 0.14 |
| **Gender** | 0.077 | 0.188 | 0.41 | 0.6835 |

Supplementary Table S2. Multiple regression predicting child's S-IgA (Z-scores)

| | **Estimate** | **Std.error** | **t value** | **P** |
|---|---|---|---|---|
| **Group** | **-0.28** | 0.28 | -1 | 0.32 |
| **birthweight** | **0.0001** | **0.0004** | 0.25 | **0.80** |
| **Mother age** | -0.004 | 0.03 | -0.15 | 0.88 |
| **Mother education (above average)** | 0.05 | 0.13 | 0.39 | 0.70 |
| **Household income** | **-0.13** | 0.33 | -0.4 | 0.70 |
| **Father Education** | -0.06 | 0.14 | -0.41 | 0.68 |
| **Child's age** | **0.09** | **0.17** | 0.53 | 0.59 |
| **Gender** | **-0.23** | **0.28** | -0.82 | 0.42 |

Supplementary Table S3. Multiple regression predicting child's OT (Z-scores)

| | **Estimate** | **Std.error** | **t value** | **P** |
|---|---|---|---|---|
| **Group** | -0.20 | 0.29 | -0.69 | 0.49 |
| **birthweight** | **-0.00002** | **0.00047** | **-0.05** | **0.96** |
| **Mother age** | -0.04 | 0.03 | -1.3 | 0.20 |
| **Mother education** | -0.02 | 0.14 | -0.14 | 0.90 |
| **Household income** | **-0.11** | 0.34 | -0.33 | 0.75 |
| **Father Education** | -0.01 | 0.15 | -0.05 | 0.96 |
| **Child's age** | **0.02** | **0.17** | 0.097 | 0.92 |
| **Gender** | **-0.55** | **0.29** | -1.92 | 0.06 |

Supplementary Table S4. Comparisons between groups in main study variables, raw data

| | KC | Control | t-test |
|---|---|---|---|
| **Child depression/anxiety** | -0.04 (0.86) | -0.01 (0.73) | $t_{(75)}$=-0.155, *p=0.87* |
| **Child OT** | 3.11 (0.20) | 3.16 (0.25) | $t_{(78)}$=-0.442, *p=0.66* |
| **Child s-IgA** | 6.18 (0.47) | 6.34 (0.53) | $t_{(70)}$=-1.411, *p=0.16* |
| **Child Focused attention** | 4.19 (0.39) | 3.82 (0.64) | $t_{(78)}$=3.157, ***p=0.002*** |
| **Child EF score** | 111.51 (10.96) | 101.06 (11.02) | $t_{(72)}$=4.085, ***p=1.129e -4*** |
| **Mother depression/anxiety** | -0.27 (0.51) | 0.30 (1.08) | $t_{(78)}$=-1.43, *p=0.157* |
| **Mother depression/anxiety (infancy)** | -0.19 (0.71) | 0.08 (0.71) | $t_{(78)}$=-3.580, ***p=5.951e -4*** |
| **Dyadic synchrony infancy** | 2.66 (0.87) | 2.23 (0.96) | $t_{(77)}$=2.077, ***p=0.041*** |
| **Dyadic synchrony childhood** | 3.49 (0.70) | 3.13 (0.80) | $t_{(76)}$=2.084, ***p=0.040*** |
| **Dyadic synchrony adulthood** | 3.95 (0.74) | 3.53 (0.77) | $t_{(76)}$=2.389, ***p=0.017*** |
